# The influence of hydrocarbon generation on the sealing capability of mudstone caprock rich in organic matter

**DOI:** 10.1038/s41598-024-51960-5

**Published:** 2024-01-22

**Authors:** Wei Yang, Yadong Bai, Xiujian Sun, Jiangong Wang, Guohui Long, Hongzhe Li, Mingtao Zuo, Haipeng Li

**Affiliations:** 1 Research Institute of Petroleum Exploration and Development-Northwest (NWGI), PetroChina, Lanzhou, 730020 China; 2 Research Institute of Exploration and Development, PetroChina Qinghai Oilfield Company, Dunhuang, 736202 China

**Keywords:** Solid Earth sciences, Energy science and technology

## Abstract

To investigate the sealing capability of mudstone caprock during the evolution of organic matter (OM)-rich mudstone, a series of hydrous pyrolysis experiments were first conducted to examine the impact of hydrocarbon generation. The pore type, pore structure, porosity, and gas breakthrough pressure of pyrolytic residual samples were analyzed by field emission scanning electron microscopy, low pressure nitrogen adsorption measurements, porosimetry, and gas breakout core experiments. To model the environment at different depths, these six experiments on hydrous pyrolysis were performed at different temperatures, lithostatic pressures, and hydrodynamic pressures, while other experimental factors such as the original sample, heating time, and rate were kept constant. The results showed that during the thermal evolution process, hydrocarbons were generated from OM in mudstone, resulting in the formation of pores within the OM. Organic acids produced by hydrocarbon generation effectively dissolved minerals, leading to the creation of numerous dissolution pores. Changes in pore type led to changes in pore structure and porosity. The volume of micropores and macropores showed an increasing trend before reaching a Ro value of 1.41%. However, after passing this threshold, they began to decrease. The volume of mesopores showed a decreasing trend before reaching a Ro value of 1.32%. After 1.32%, they began to increase. The porosity was mainly affected by the pore volumes of the mesopores and macropores. The porosity exhibited two peaks: the first occurred at a Ro value of 0.72%, with a porosity level of 4.6%. The second occurred at a Ro value of 1.41% and a porosity level of 10.3%. The breakthrough pressure was a comprehensive reflection of these influences, and its trend exhibited a negative correlation with porosity (R^2^ = 0.886). For two high values of porosity, the breakthrough pressure corresponded to two low values. Smaller values of the breakthrough pressure indicated a poorer sealing capability of the mudstone caprock. Overall, hydrocarbon generation in the mudstone affected the sealing capability. The mudstone in the studied area exhibited good sealing at Ro below 1.32%. However, once above the 1.32% threshold, the fluctuations of the breakthrough pressure values exhibited considerable variability, requiring a comprehensive evaluation to assess its sealing capability.

## Introduction

The caprock, serving as a geological barrier for hydrocarbon reservoirs, exerts direct influence on the accumulation and preservation of both oil and gas^1^. Natural gas is small-molecule, light-weight and highly mobile, making it easier to diffuse and disperse than oil^2,3^. Mudstone serves as the dominant caprock in China's natural gas reservoirs, with numerous mudstone formation exhibiting high OM content and acting as both caprock and source rock for oil and gas^4^. Typically, mudstone containing more than 2% OM is referred to as organic-rich mudstone^5^. Dispersed, fine-grained porous organic matter in mudstone is typically distributed in an inorganic matrix, resulting in a large number of OM pores after hydrocarbon generation^6–8^. Organic micropores and capillary pores smaller than 100 nm constitute the main pore volume in mudstone ^9–11^.

Several scholars have found that the presence of OM significantly affects the sealing properties of mudstone caprock. Zheng et al.^12^ focused on investigating the impact of hydrocarbon generation from OM on caprock sealing. They propose that during the initial stages of burial, there was a sudden increase in organic acid concentration within the formation, which leads to substantial erosion of feldspar and rock debris^13^. As a result, particle support is significantly reduced and pore fluid overpressure develops in the mudstone, thereby enhancing caprock sealing^14–16^. However, as OM enters the hydrocarbon generation window phase, a considerable amount of it is converted into hydrocarbons resulting in increased porosity and deterioration of caprock sealing. Meng^17^ conducted extensive studies of a large set of dark mudstone, which serves both as caprock and a significant source rock, found in the Damintun Sag of the Liaohe River. The porosity of the mudstone gradually decreases with increasing burial depth and compaction; however, the breakthrough pressure exhibits two prominent peaks with burial depth that do not correspond to changes in porosity and permeability^18^. Jarvie et al.^19^ reported that mudstone with an OM content of 7% consumed 35% of the available carbon during hydrocarbon generation, resulting in a significant increase in porosity of 4.9%. The hydrocarbon generation in organic matter-rich mudstone caprock inevitably exerts a significant influence on their sealing capability, particularly in the lower and upper sections where numerous micropores are generated through processes driven by OM^20^. This direct impact plays a crucial role in the formation of natural gas reservoirs^21^.

So far, although some scholars have focused on mudstone caprocks with hydrocarbon generation capacity, the results are still not systematic. A full investigation of the effect of OM-derived hydrocarbon generation on the sealing capability of thermally mature mudstone caprock is yet to be conducted. The primary factors influencing the variability in mudstone caprock sealing capability encompass pore structure, pore type, and porosity^22,23^. In response to the aforementioned concerns, this paper presents pyrolysis experiments conducted on mudstone caprock rich in OM, with a specific focus on investigating the changes in pore structure, porosity, pore type, and breakthrough pressure at different stages of hydrocarbon generation as thermal maturity increases. The aim of this study is to enhance our comprehension of the evolutionary patterns governing the sealing capability of mudstone caprock, systematically assess the primary controlling factors influencing the sealing capability of organic matter-rich mudstone, and further improve and refine evaluation methodologies for assessing mudstone caprock.

## Experiment

### Sample

**Figure 1:**
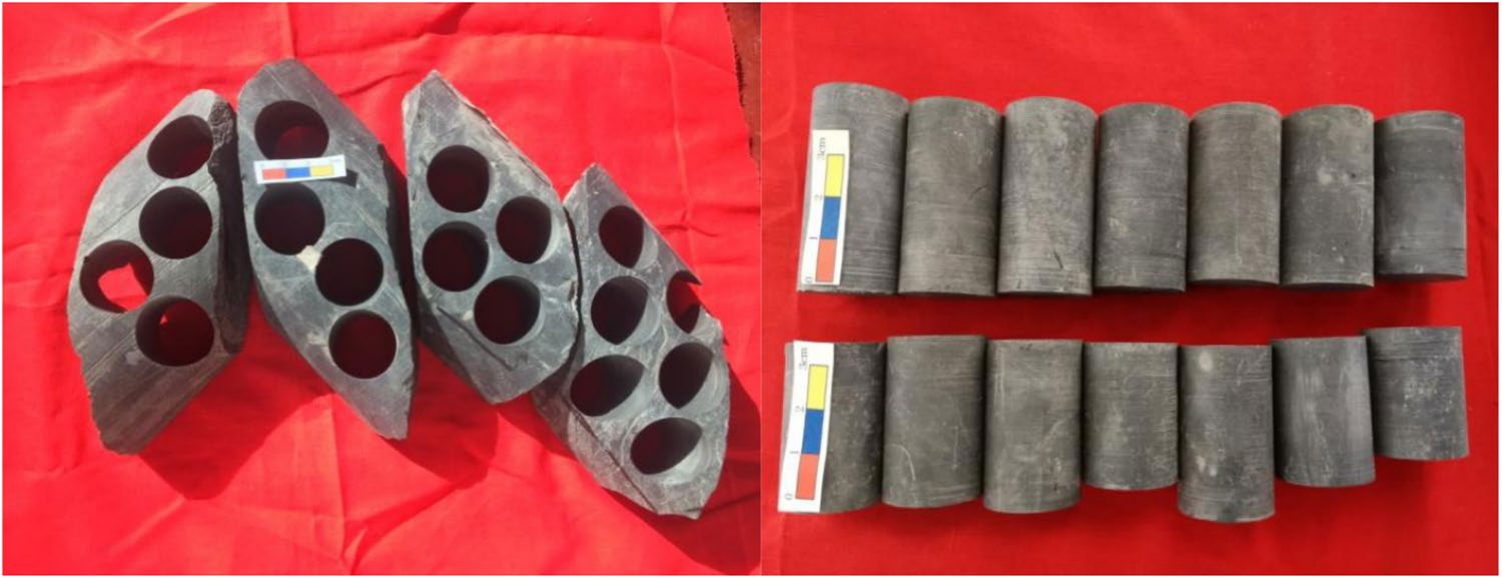
The original core samples and drilling core cylindrical samples with a 25 mm diameter.

The block mudstone core samples were collected from S86 well in Huxishan Formation, Jurassic, Qaidam Basin, western China. Before pyrolysis, the block samples were continuously drilled into cylindrical samples with a diameter of 25 mm and a height of 3–6 cm (Fig. 1). The sample was OM-rich, with a TOC of 4.71%, and the vitrinite reflectance (Ro) is about 0.59%, which indicated that the thermal maturity of the OM in this mudstone sample was immature to low mature.

### Pyrolysis experiments

**Figure 2:**
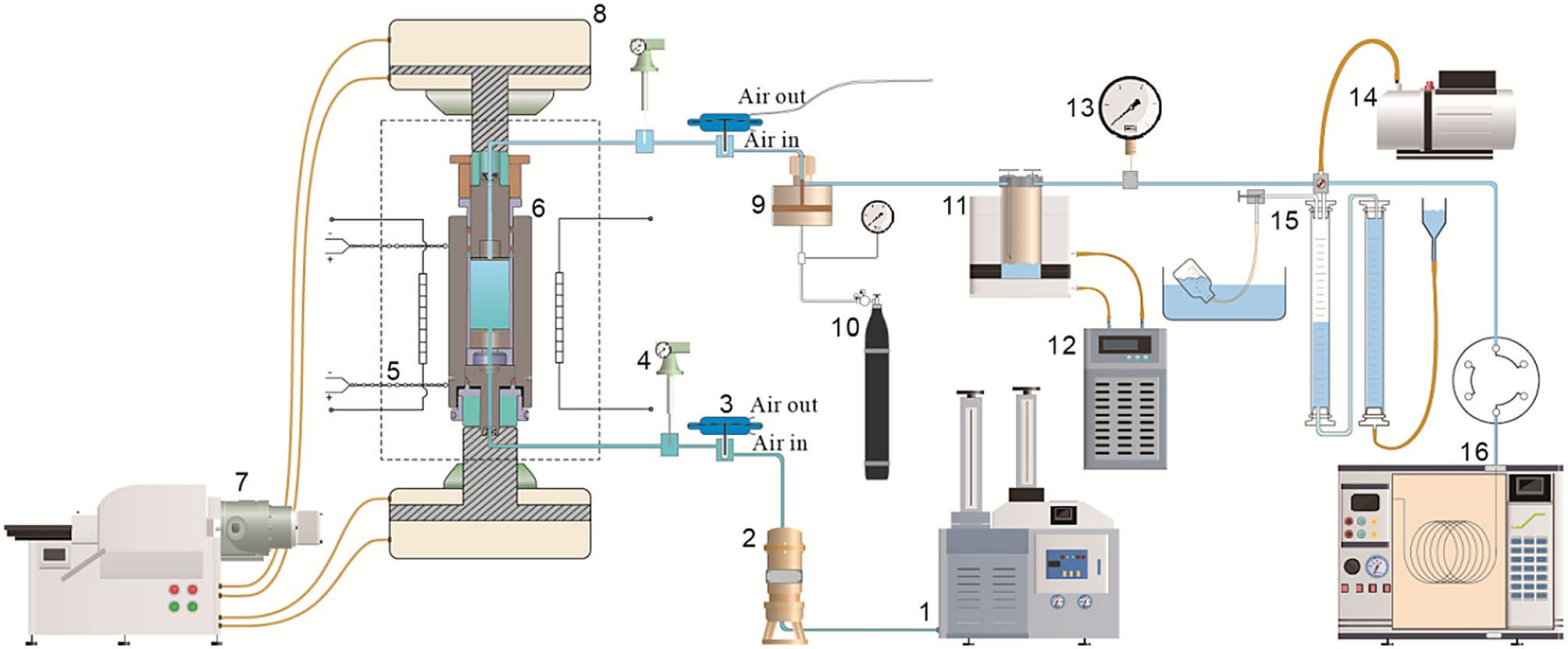
Schematic of pyrolysis equipment (not to scale)^26^. 1. High-pressure pump; 2. high-pressure piston container; 3. high-pressure pneumatic valve; 4. pressure transmitter; 5. heating furnace and thermocouple; 6. autoclave; 7. hydraulic control system (with a plunger pump); 8. hydraulic cylinder; 9. back-pressure valve; 10. nitrogen gas cylinder (with reducing valve); 11. gas and liquid collector and cold trap; 12. cooling water circulation machine; 13. vacuum gauge; 14. vacuum pump; 15. gas collecting pipe; 16. gas chromatograph equipped with a six-pass valve feeder.

The pyrolysis experiments were conducted in a WYMN-3 high-temperature and high-pressure simulation instrument (Fig. 2), which consists of a software control system (computer) and a hardware performance system (instrument)^24^. Computer programs is used to set experimental conditions and collect data. The performance system includes the following secondary systems: reaction (autoclave), heating, hydraulic control (control of the lithostatic pressure), fluid replenishment (add deionized water and control of the dynamic water pressure) and collection (store pyrolysis products) systems^24–26^.

Based on the measured geological burial history, lithostatic pressure and fluid pressure were designed to be coupled. The lithostatic pressure is provided by the hydraulic control system and is used to simulate the pressure of overlying rock underground, which can be seen as a contributor to diagenesis^24,25^. The fluid pressure is produced by the action of hydrous pyrolysis in the confined space of the sample cell. The range of fluid pressure fluctuation is between the minimum and maximum pressure coefficients (Cp) due to the intermittent opening of the sealing layer. When the fluid pressure in the sample cell reached the pressure limit, the back-pressure valves opened to discharge hydrocarbon into the gas and liquid collector. The fluid in the autoclave is discharged into the cold trap that is designed to collect the product. The gas generated is collected by the method of drainage water. When the fluid pressure is reduced to the minimum value, the back-pressure valves automatically close. Thus, pyrolytic systems are in a dynamical evolution between an open and a closed system, which is closer to natural geology.

Before the pyrolysis experiments, the sample was drilled to core columns with a diameter of 25 mm and a height of 3–6 cm, which is a suit size for the sample cell. The sample cell with the core column sample was placed in the autoclave and deionized water was used as the pressure medium with a liquid-rock ratio about 0.8–1.2. Then the experimental procedure was as follows: (1) The leakage test was carried out, and the experimental device was vacuumed (2) Six temperature points were conducted on six columned cores separately; the temperatures used were from 250 to 500 °C with an interval of 50 °C. Samples were heated from room temperature to the target temperature in 2 h and remained at the target temperature for 48 h. (3) According to the simulated depth of the sample, lithostatic pressures were set from 74 to 135 MPa, and the fluid pressures were set from 29 to 54 MPa by adding deionized water from the high-pressure pump to the sample cell (Table 1). After every pyrolysis experiment was completed, the gas and fluid were collected respectively, and the cylindrical solid residue was recycled and prepared for other analysis.

**Table 1:** Parameters set by pyrolysis experiments according to natural geology.

Pyrolysis temperature (°C)	Heating rate (°C/h)	Constant temperature time (h)	Simulated depth (m)	Lithostatic pressure (/Mpa)	Fluid pressure (/MPa)		
					Ref	Min	Max
250	2	48	3000	74	29	26	35
300	2	48	3500	86	34	31	41
350	2	48	4000	98	39	35	47
400	2	48	4500	110	44	40	53
450	2	48	5000	123	49	44	59
500	2	48	5500	135	54	49	65

Simulation depth: similar to the depth of the real underground.

Lithostatic pressure = ρ_s_gh, ρ_s_ = 2.4 g/cm^3^, g = 9.8N/kg, h = Simulation depth.

Fluid pressure = ρ_w_gh·Cp, ρ_w_ = 1.0 g/cm^3^, Cp = 0.9–1.2.

### Pore appearance

In order to observe the characteristics of pores, the solid samples after pyrolysis experiment were first polished by argon ion to obtain a smooth, fresh rock surface. Then the surface of the sample was gold-plated with a thickness of about 15 nm to improve the image quality, and then observed by field emission-scanning electron microscopy (FE-SEM).

### Analysis of pore structure

Low temperature nitrogen adsorption measurements on the solid residues were conducted on an ASAP 2020 HD88 surface area analyzer. Before the experiment, the samples were crushed to 60 mesh and degassed at 150 °C for 8 h in a vacuum chamber. The high purity nitrogen was injected and isothermal physical adsorption–desorption were performed at 77 K with a relative pressure range of 0.01–0.998. Then the data of the adsorption–desorption isotherm curves were obtained. The effective average pore diameters range from 0.35 to 500 nm, with the capability of measuring minimum specific surface area down to 0.0005 m^2^/g and detecting minimum pore volume as low as 0.0001 cm^3^/g.

### Porosity

The effective porosity was determined using the PoroPDP-200 overburden porosity measurement instrument, employing 3–6 cm cylindrical rock core samples. Porosity measurements were conducted based on the helium gas expansion principle, utilizing Boyle’s law for calculation. The instrument employed a high-precision pressure sensor with a pressure range of 0–100 psi and an accuracy of ± 0.1% of full scale. The measured porosity ranged from 0.01 to 40%.

### Analysis of breakthrough pressure

The gas breakthrough core experiments were conducted in a SCMS-C3 fully automatic multi-parameter core analyzer. According to the characteristics of dense core, the sample is firstly vacuumed, and then saturated with formation water (CaCl_2_-type). The core sample was placed in a core holder, the upstream side was connected to a high-pressure gas source, and a hose was attached at the water-submerged exit to observe the bubble release. It is essential to ensure proper equipment connection and purge any residual gas from the pipe before proceeding. To prevent leakage, an initial confining pressure of 8–10 MPa was applied. The gas pressure started at 0.01 MPa and increased by 15% of the previous pressure every 10 min. When a continuous bubble emerged from the outlet, it indicated that the rock sample had reached its breaking point. Record the breaking pressure and time during the experimental process.

## Results and discussion

### Pyrolysis products

The pyrolysis of the OM resulted in a gradual decrease in the measured TOC content of solid residues from the initial 4.71–2.25%. Simultaneously, the calculated values of Ro (based on Sweeney and Burnham's model in 1990^27^ and Tang et al.'s model in 1996^28^) exhibited a progressive increase from 0.68 to 1.61% (Table 2).

**Table 2:** The values of increased Ro and porosity based on decreased TOC with the increasing pyrolysis temperature.

Pyrolysis temperature (°C)	Ro (%)	TOC (%)	Porosity (%)
Unheated sample	0.59	4.71	2.3
250	0.68	4.35	2.8
300	0.72	3.68	4.6
350	0.95	3.46	3.4
400	1.32	2.92	5.1
450	1.41	2.62	10.3
500	1.61	2.25	6.2

**Figure 3:**
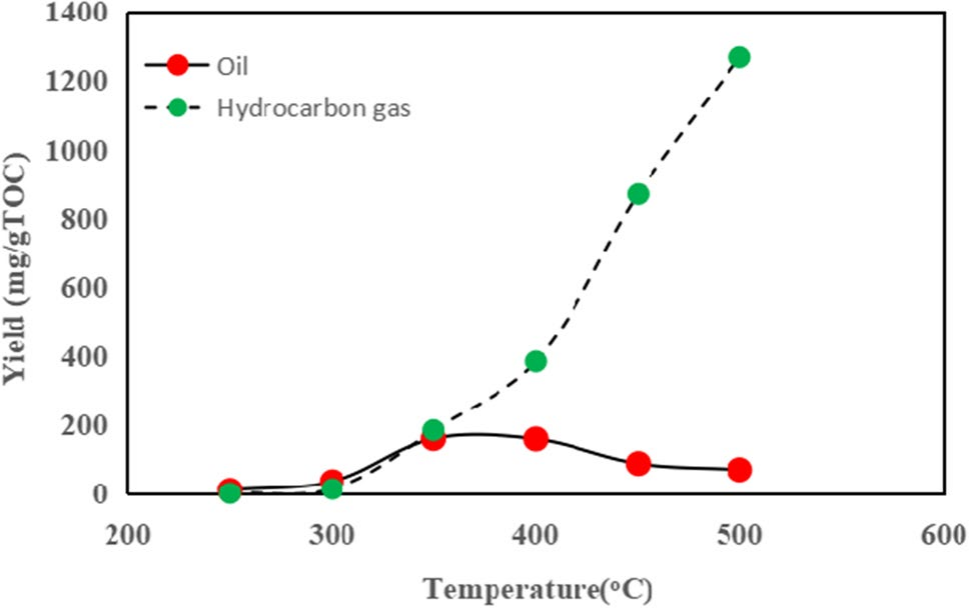
The yields of oil and hydrocarbon gas in the pyrolysis.

The gas and oil yields are shown in Fig. 3. Oil yield represents the combined amount of expelled and residual oil. We can categorize the evolution of oil and gas into three distinct stages. Firstly, within the temperature range of 250–300 °C, with a Ro value ranging from 0.68 to 0.72%, there was minimal generation of both oil and gas. Secondly, during the temperature range of 350–400 °C, with a Ro value between 0.95 and 1.32%, this phase signified substantial production of oil followed by significant quantities of gases being generated subsequently. The initiation point for the Ro values in the oil window was approximately at 0.95%. Lastly, spanning from temperatures between 450 and 500 °C, with a Ro value ranging from 1.41 to 1.61%, there was a decline in oil yield due to thermal cracking leading to conversion from oil to gas. Concurrently, an increase in the gas yield was also observed under pyrolytic experimental conditions. Data on yields for both oil and gas indicate that hydrocarbons are formed through OM during burial in mudstone caprock.

### Pore types evolution after pyrolysis

**Figure 4:**
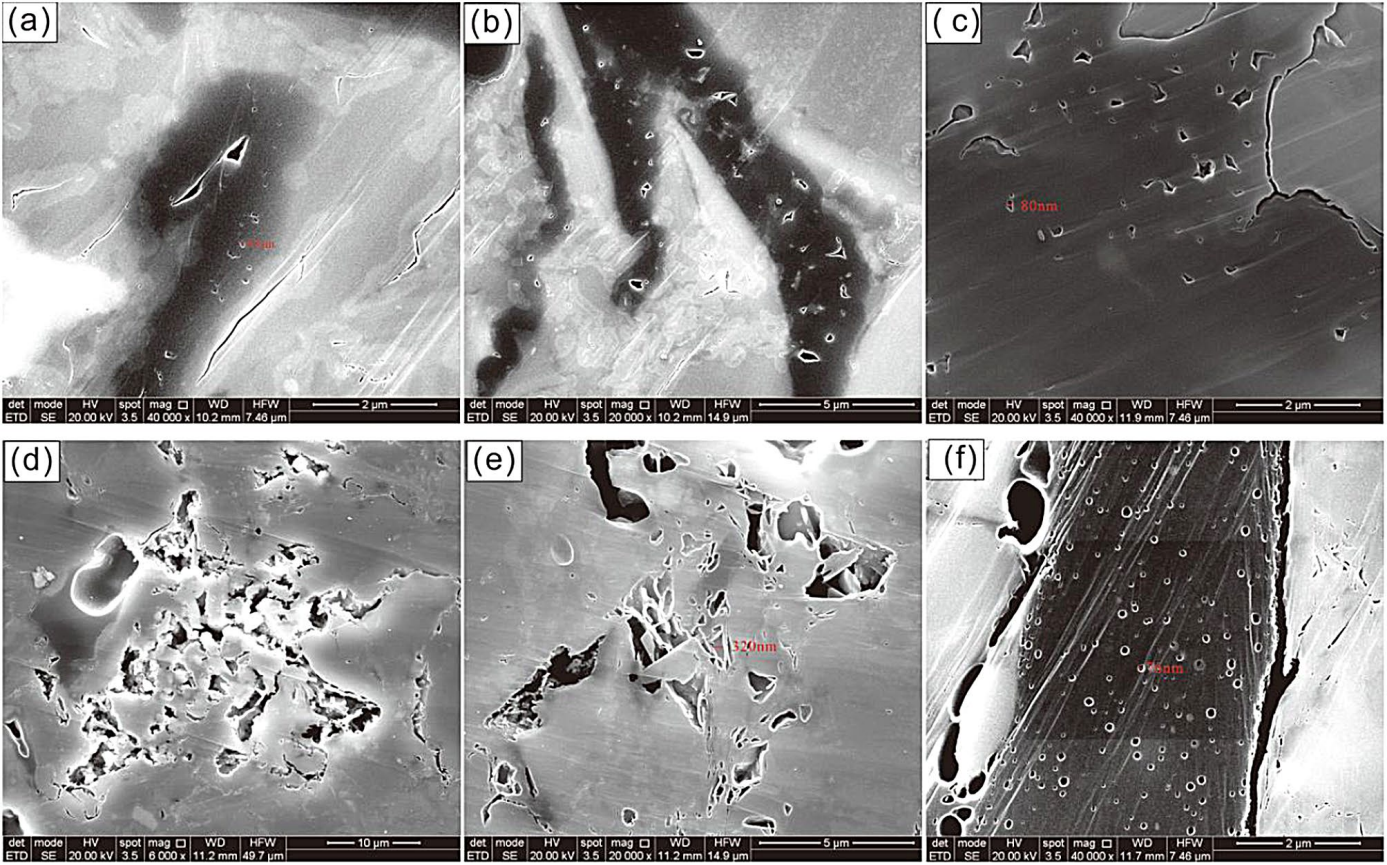
Microscopic characteristics of pyrolysis-induced pores by FE-SEM (**a**) Ro:0.68%; (**b**) Ro:0.72%; (**c**) Ro:0.95%; (**d**) Ro:1.32%; (**e**) Ro:1.41%; (**f**) Ro:1.61%.

The alteration of the pore type constitutes a crucial factor affecting the variation in porosity. Examination of the cylindrical solid residues after thermal pyrolysis using FE-SEM revealed variations in the pore morphology of the mudstone throughout its thermal evolution. In the generation of hydrocarbons, OM not only generates hydrocarbons but also forms organic acids, which also facilitate the dissolution of specific minerals such as carbonate minerals. Moreover, the resulting percolation pores contribute significantly to the formation of crucial pores during the process of hydrocarbon generation from organic matter. When the OM was in the low maturity to mature stage (Ro < 0.8%), bubble-like organic pores were dispersed within them (Fig. 4a, b), while shrinkage cracks developed along the edges of the OM. The increase in porosity was relatively gradual, but facilitated caprock closure. After reaching a Ro of 0.95%, the OM exhibited substantial hydrocarbon generation, leading to the migration of a portion of the resultant oil and gas from the pore space. Subsequently, two distinct alterations occurred within the pores. First, residual pores of hydrocarbon generation were created within the OM, leading to a reduction in the volume of the OM and the formation of contracted organic pores between the OM and the skeletal minerals. (Fig. 4c). Secondly, the expulsion of organic acids during hydrocarbon generation enhanced mineral solubility, resulting in the dissolution of unstable minerals and the subsequent development of numerous secondary pores, thereby significantly augmenting the porosity of the mudstone (Fig. 4d, e). At this stage, the development of the pores did not favor efficient sealing of the oil and gas. By the time OM evolution reached the high-maturity phase (Ro > 1.3%), a significant portion of it had undergone conversion into oil and gas, resulting in an essential depletion of hydrocarbon generation potential. The extent of hydrocarbon conversion during this phase was relatively limited compared to previous stages, thus limiting the increase in porosity (Fig. 4f). According to the IUPAC classification^29^, pores with pore size between 0 and 2 nm are defined as micropore, pores with pore size between 2 and 50 nm are defined as mesopore, and pores with pore size greater than 50 nm are defined as macropore. The pores of the OM generated during hydrocarbon generation exhibit mainly micro- and mesopores, whereas the dissolution pores resulting from the expulsion of organic acids exhibit predominantly macropores. These dissolution pores significantly contribute to porosity while simultaneously compromising the sealing capability of the mudstone when serving as caprock.

### Pore structure evolution in the pyrolysis

**Figure 5:**
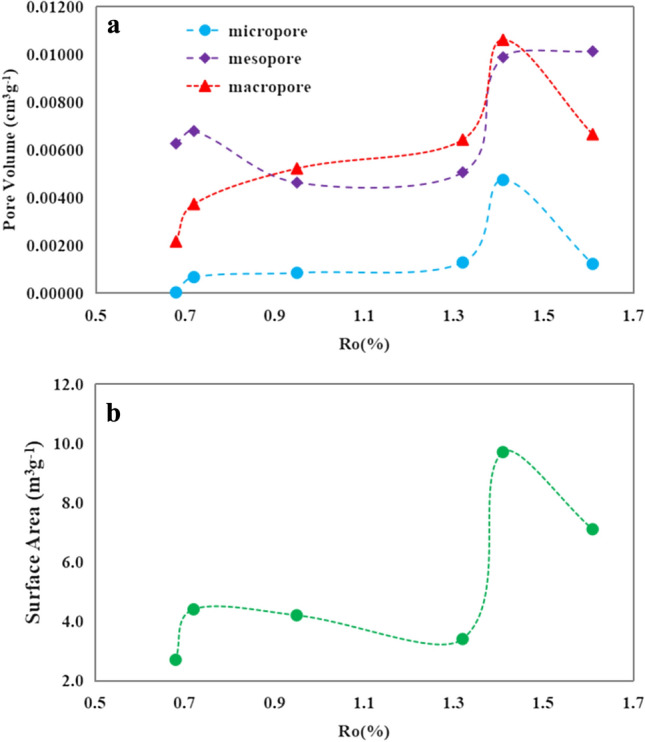
Pore volume (**a**) and surface area (**b**) distributions obtained from N_2_ adsorption experiments at different pyrolysis stages.

The pore size, distribution, and specific surface area were obtained from nitrogen (N_2_) adsorption experiments. When Ro reached 0.68%, the OM has just initiated pyrolysis, resulting in the formation of hydrocarbons. During this stage, the micropore volume was relatively small, while the mesopore volume dominated (Fig. 5a). The pyrolysis conditions primarily affected the residual mesopores. As the pyrolysis strength of the OM increased, the pore development continued. At 0.95%Ro, the macropore volume exceeded the mesopore volume and the micropore volume occupied the lowest proportion. This trend continued until Ro reached 1.41%. Subsequently, the migration of oil and gas from the pores reduces the resistance to overlying compaction, leading to a reduction in the macropore volume and a reduction in the blockage/reduction of the micropore space. Simultaneously, the contraction of the macropore caused an increase in the volume of the mesopore (Fig. 5a). The pore structure plays a pivotal role in determining the sealing capability^30–33^. Mudstone typically exhibits internal heterogeneity, leading to diverse pore development and influencing its sealing capability^34–37^. In general, smaller pore sizes indicate denser rocks with enhanced closure capabilities.

The pore size directly impacts the specific surface area of the sample, with micropores and mesopores making significant contributions to its overall value. A larger specific surface area enhances the adsorption capacity of the sample, leading to a thicker gas adsorption layer on the pore walls^38,39^. When the Ro value was 0.68%, the cumulative specific surface area was low due to the limited formation of organic pores in the sample and the lesser development of micropores. As the degree of thermal evolution increased, the optimal development of micropores and mesopores occurred at Ro of 1.41%, resulting in a significant increase in the cumulative specific surface area, possibly up to approximately 10 cm^3^/g (Fig. 5b). Subsequently, the cumulative specific surface area showed a decreasing trend, which is attributed to the alteration of the pore structure. Overall, Ro up to 1.41% resulted in a limited cumulative specific surface area of no more than 5 cm^3^/g, which adversely affected gas adsorption and hindered the formation of hydrocarbon concentration closures.

### Porosity evolution in the pyrolysis

Chen et al.^40^ has studied the porosity changes during thermal hydrocarbon generation from OM, revealing an initial decrease followed by an increase in porosity within the Ro range of 0.6–2.0%. This phenomenon can be attributed to the filling of organic pores by oil and gas, as well as asphalt cracking occurring in the oil generation window. In order to address the requirements of oil and gas exploration, this study refines the evaluation of caprock sealing by considering variations in porosity during hydrocarbon generation based on previous research.

**Figure 6:**
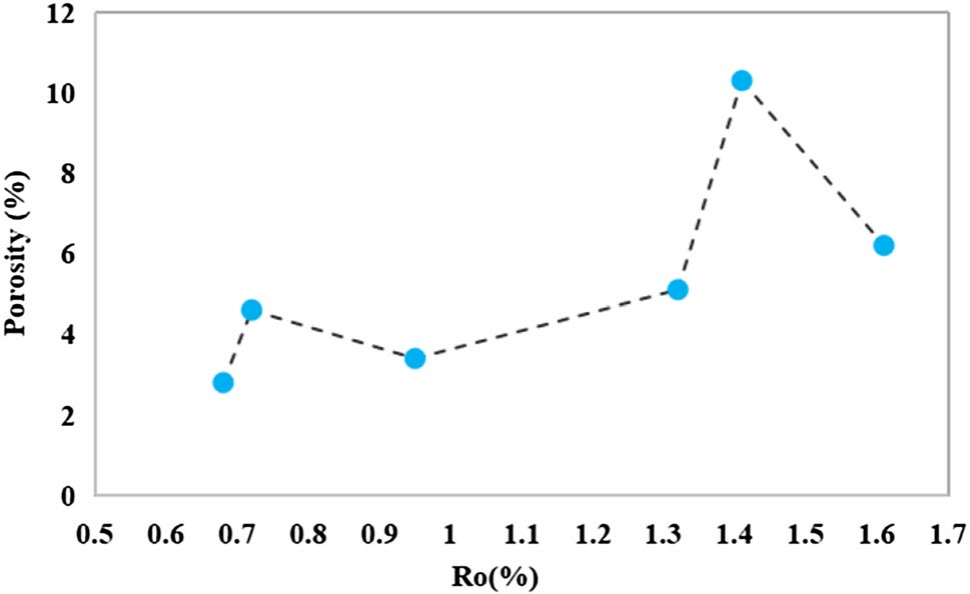
Porosity of the samples after pyrolysis.

Porosity tests of the cylinders after the experiment revealed two distinct peaks in the porosity curve following hydrocarbon production from the OM. The first peak in porosity occurred at 0.72% Ro in the range 0.68–0.95%, followed by a subsequent decrease in porosity (Fig. 6). Subsequently, a secondary peak in porosity occurred at 1.41% of Ro within the interval of 0.95–1.61%, followed by another decrease in porosity. When the porosity reached 10.3%, the mudstone was extremely unfavourable for use as a covering. In terms of overall trends, the variability in the porosity of the mudstone caprock exhibited relative stability below 1.3% Ro, indicating favorable conditions for oil and gas containment purposes. However, after reaching a maturity stage characterized by hydrocarbon generation evolution, a significant shift in porosity occurred in conjunction with oil and gas production, necessitating a comprehensive test analysis to assess the scalability of the caprock during this stage.

The evaluation of caprock sealing capability necessitates the consideration of porosity as a crucial factor^41–44^. The observed variability in porosity can be attributed to a combination of factors including increased porosity due to hydrocarbon production, enhanced porosity due to dissolution, and reduced porosity due to compaction and cementation. When Ro was below 0.72%, the hydrocarbon generation from OM exhibited a moderate level. The conversion of the OM-derived hydrocarbons resulted in their accumulation within the pore spaces, leading to pore blockage and subsequent pressure increase, which effectively counteracted the overlying compaction. When the pore pressure initially reached the threshold for hydrocarbon expulsion, a portion of the hydrocarbon was expelled, thereby intensifying the compaction in the overlying strata and resulting in a subsequent decline in porosity. The evolution of hydrocarbon generation is an ongoing process. The porosity reached its second peak when the oil and gas content in the pores continued to increase, leading to the attainment of a second threshold for hydrocarbon expulsion. The subsequent expulsion of the oil and gas resulted in an enhanced compaction effect, which further reduces the porosity. The closure of the mudstone caprock is influenced by any alteration of the porosity. Similar to the transition of source rocks from immaturity to maturity and high maturity, caprock can also undergo a progression from ineffectiveness to effectiveness and back to ineffectiveness.

### Response of the breakthrough pressure evolution to the pyrolysis process

**Figure 7:**
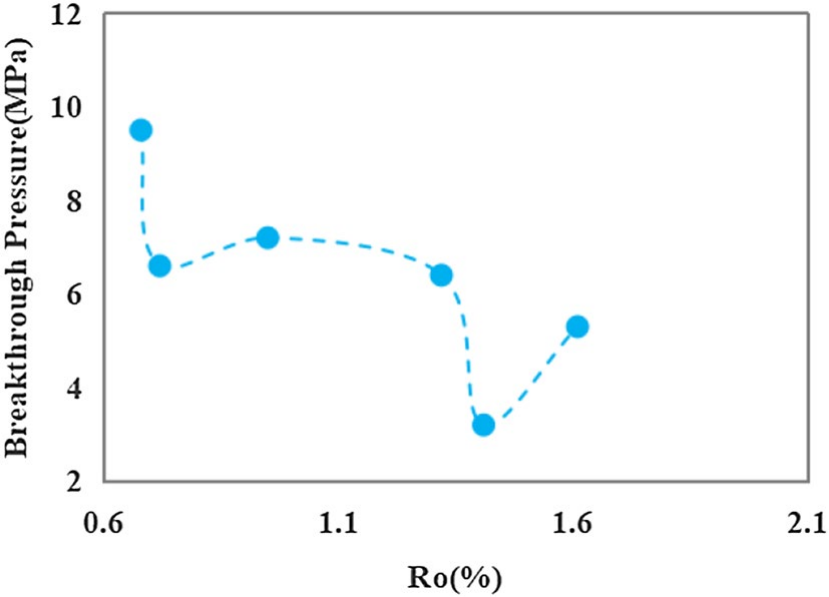
Breakthrough pressures of the samples after pyrolysis.

The breakthrough pressure, which serves as a key parameter for caprock assessment, exhibits the utmost efficacy^45^. It directly reflects the sealing capability of the caprock and is influenced by significant factors such as the pore type, pore structure and porosity within the mudstone sample. The values of the breakthrough pressure at different temperatures are presented in Table 3. It decreased to 9.5 MPa when Ro reached 0.68%, indicating a discernible impact of hydrocarbon generation from OM on caprock compared with the unheated sample (Fig. 7). At a Ro of 0.72%, the breakthrough pressure continued to decrease, accompanied by the emergence of the initial low peak value of 6.6 MPa. The breakthrough pressure value increased at a Ro of 0.95%, most likely attributed to the expulsion of hydrocarbon and sample compaction. The breakthrough pressure values exhibited slight variability across the three thermal maturation phases, with pressures above 6 MPa consistently observed throughout these phases.

**Table 3:** The breakthrough pressure of samples after the pyrolysis.

Pyrolysis temperature (°C)	Ro	Breakthrough pressure (MPa)
Unheated sample	0.59	10.9
250	0.68	9.5
300	0.72	6.6
350	0.95	7.2
400	1.32	6.4
450	1.41	3.2
500	1.61	5.3

A second low pressure of 3.2 MPa was observed when the Ro value reached 1.32%, which also represented the lowest pressure during the entire pyrolysis. At this stage, the sample exhibited its highest porosity, with corresponding maximum pore volume for micropores, mesopores, and macropores (Fig. 5a). Subsequently, as Ro reached 1.61%, there was an increasing trend in breakthrough pressure values indicating extensive crude oil cracking. The consumption of crude oil within the pores led to compaction dominance and an overall reduction of the pore volume. Consequently, breakthrough pressure values increased and sealing ability was enhanced. Mudstone samples demonstrating hydrocarbon generation ability exhibited higher breakthrough pressure values and enhanced sealing capability during both early maturation stages and throughout maturity (Ro: 1.3%). However, during the high-maturity stage of mudstone, significant changes in the sealing capability occurred due to simultaneous hydrocarbon generation, corrosion, and compaction processes. A full assessment of the sealing capability of the mudstone is necessary at this stage.

**Figure 8:**
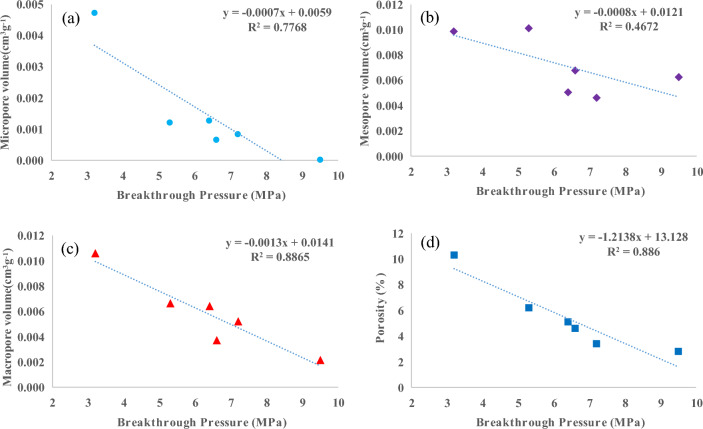
The correlations between breakthrough pressure and micropore volume, mesopore volume, macropore volume, as well as porosity.

The correlation analysis of the measured data from the simulated samples reveals a negative relationship between the breakthrough pressure and the pore volume of micropores, mesopores, and macropores in mudstone (Fig. 8). Notably, higher correlation coefficients (R^2^) are observed for micropores and macropores. The cumulative pore volume of micropores, mesopores, and macropores constitutes the porosity of the sample, with the proportion of mesopores and macropores being the dominant factor. A negative correlation was found between breakthrough pressure and porosity, with a significantly higher correlation coefficient of 0.886 indicating that as porosity increases in the sample, corresponding breakthrough pressure decreases thereby compromising the caprock sealing properties. Based on the comprehensive analysis presented above, it is believed that the sealing efficacy of organic-rich mudstone caprock may not have remained constant throughout the burial process. When the Ro value in the study area was below 1.3%, its sealing capability exhibited a relatively favorable state, followed by a transition process from inefficiency to effectiveness.

The primary objective of the present experiment was to simulate the development of organic pores in OM and the associated acid dissolution and porosity destruction during pyrolysis of mudstone for hydrocarbon generation. However, limited simulations have not explored the effect of cementation on caprock properties due to the brevity of experimental studies. Nevertheless, the overall trajectory of the caprock sealing capability evolution is of significant interest.

## Conclusions

The sealing capability of organic-rich mudstone caprock varies throughout the stages of hydrocarbon generation as the burial depth increases. The pore type, pore structure, and porosity of the mudstone samples are the primary factors that affect the sealing capability of the mudstone caprock. By pyrolysis experiments, the following conclusions were obtained.

1. Hydrocarbon generation from OM was limited when the Ro was below 0.95%, resulting in the development of primary pores with mesopore sizes and sporadic OM nanopores in the sample. However, this did not significantly affect the total porosity or sealing capability of the caprock. When Ro reaches 0.95%, an enhancement in the hydrocarbon production strength of the OM was observed, accompanied by the significant appearance of numerous organic pores and dissolution pores resulting from the dissolution of soluble minerals by organic acids. Nevertheless, this phenomenon posed a challenge to caprock sealing. When Ro exceeded 1.32%, significant amounts of organic pores, corrosion pores, and microfractures developed in the mudstone, potentially having a considerable effect on the overall caprock sealing properties.
2. The hydrocarbon conversion of the OM resulted in a relatively small fraction of micropore volume developing within the overall pore volume of the mudstone sample, with the highest proportion observed at 1.41% Ro. The mesopore volume initially increased, followed by a decrease and a subsequent increase. Meanwhile, the macropore volume reached its maximum at 1.41% Ro before declining due to hydrocarbon expulsion and compaction. A larger pore volume was found to be associated with poorer sealing properties of the mudstone.
3. Two peaks of porosity were observed in the cylindrical sample after hydrocarbon generation. A minor peak in porosity occurred at 0.72% Ro, indicating the presence of pyrolytic products from a lower maturity stage within the pores. At a Ro value of 1.41%, the maximum hydrocarbon generation was achieved and the porosity reached its peak. It is worth noting that the high porosity did not facilitate efficient sealing by the caprock.
4. The unheated sample exhibits a breakthrough pressure value of 10.9 MPa. Following pyrolysis, the breakthrough pressure gradually decreased until Ro reached 1.32%. At a Ro of 1.41%, the breakthrough pressure reached its minimum value due to pore development. As the OM continued to crack, the oil within the pores underwent cracking as well, leading to compaction dominance and enhanced caprock properties. The sealing properties of mudstone rocks with hydrocarbon production capacity varied across burial depths. Therefore, a full assessment is necessary to assess the sealing properties of the mudstone after reaching a Ro of 1.3%.

## Data Availability

The datasets used and/or analysed during the current study available from the corresponding author on reasonable request.

## References

[1] Ovcharenko AV (2007). Caprocks in hydrocarbon fields. Lithol. Miner. Resour..

[2] Hao SS (2000). Geophysical properties of cap rocks in Qiongdongnan Basin, South China Sea. Mar. Pet. Geol..

[3] Huang ZL (2007). Diffusion mechanism and quantitative model of microleakage in caprocks. Nat. Gas. Geosci..

[4] Fu G, Wang PY (1997). Special use of mudstone source rocks in capping oil and gas. Nat. Gas. Geosci..

[5] Zou C (2016). Shale gas in China: Characteristics, challenges and prospects (II). Pet. Explor. Dev..

[6] Bowker KA (2007). Bmett shale gas production, Fort Worth Basin: Issues and discussion. AAPG Bull..

[7] Chalmers GRL, Bustin RM (2008). Lower cretaceous gas shales in northeastern British Columbia, part I: Geological controls on methane sorption capacity. Bull. Can. Pet. Geol..

[8] Ross DJ, Bustin RM (2009). The importance of shale composition and pore structure upon gas storage potential of shale gas reservoirs. Mar. Pet. Geol..

[9] Ambrose R (2010). Newpore-scale considerations for shale gas in place calculations. SPE.

[10] Rodriguez ND, Philp RP (2010). Geochemical characterization of gases from the Mississippian Barnett Shale, Fort Worth Basin, Texas. AAPG Bull..

[11] Curtis ME (2012). Development of organic porosity in the Woodford Shale with increasing thermal maturity. Int. J. Coal Geol..

[12] Zheng CY, Zhang WD, Zhu PL (1996). Cover types and their control over hydrocabon migration and accumulation. Oil Gas Geol..

[13] Hosseini M (2018). Formation evaluation of a clastic gas reservoir: Presentation of a solution to a fundamentally difficult problem. J. Geophys. Eng..

[14] Hosseini M (2022). Capillary sealing efficiency analysis of caprocks: Implication for hydrogen geological storage. Energy Fuel.

[15] Hosseini M (2022). Assessment of rock-hydrogen and rock-water interfacial tension in shale, evaporite and basaltic rocks. J. Nat. Gas Sci. Eng..

[16] Hosseini M (2021). Neutron scattering: A subsurface application review. Earth-Sci. Rev..

[17] Meng WG (2005). Characteristics of the Paleogene caprock and influence on petroleum systems in Damintun Sag of Liaohe Depression. J. Palaeogeogr..

[18] Hosseini M, Abdolrahim J, Bahram M (2014). Determination of permeability index using Stoneley slowness analysis, NMR models, and formation evaluations: A case study from a gas reservoir, south of Iran. J. Appl. Geophys..

[19] Jarvie DM (2007). Unconventional shale gas systems: The Mississippian Barnet t shale of north-central Texas as one model for thermogenic shale gas assessment. AAPG Bull..

[20] Zhang K (2019). Vertical sealing mechanism of shale and its roof and floor and effect on shale gas accumulation, a case study of marine shale in Sichuan basin, the Upper Yangtze area. J. Pet. Sci. Eng..

[21] Krooss BM (1995). Generation of nitrogen and methane from sedimentary organic matter: Implications on the dynamics of natural gas accumulations. Chem. Geol..

[22] Hosseini M (2016). Estimation of mean pore-size using formation evaluation and Stoneley slowness. J. Nat. Gas Sci. Eng..

[23] Yang W (2020). Characterization of the weathered basement rocks in the Dongping field from the Qaidam Basin, Western China: significance as gas reservoirs. Sci. Rep..

[24] Sun LN (2015). Formation and development of the pore structure in Chang 7 member oil-shale from Ordos Basin during organic matter evolution induced by hydrous pyrolysis. Fuel.

[25] Song D (2019). Hydrocarbon generation potential and evolution of pore characteristics of Mesoproterozoic shales in North China: Results from semi-closed pyrolysis experiments. J. Nat. Gas Sci. Eng..

[26] Zhang DW (2020). The chemical kinetics of the semi-open hydrous pyrolysis system: Time series analysis of lithostatic pressure and fluid pressure. Int. J. Coal Geol..

[27] Sweeney JJ, Burnham AK (1990). Evaluation of a simple model of vitrinite reflectance based on chemical kinetics. AAPG Bull..

[28] Tang Y (1996). Modeling early methane generation in coal. Energy Fuels.

[29] Sing KSW (1985). Reporting physisorption data for gas/solid systems with special reference to the determination of surface area and porosity (Recommendations 1984). Pure Appl. Chem..

[30] Fu G (1998). Comprehensive evaluation of sealing ability of mudstone caprock. Pet. Geol. Exp..

[31] Dawson WC, Almonm WR (2002). Top seal potential of Tertiary deep-water Gulf of Mexico shales. GCAGS.

[32] Yu LJ (2011). Seal mechanism of cap rocks. Pet. Geol. Exp..

[33] Tang X (2016). Effect of organic matter and maturity on pore size distribution and gas storage capacity in high-mature to post-mature shales. Energy Fuel.

[34] Yuan YS (2011). Several discussions of sealing capacity studies of caprock. Pet. Geol. Exp..

[35] Deming D, Cranganu C, Lee Y (2002). Self-sealing in sedimentary basins. J. Geophys. Res..

[36] Lash GG (2006). Top seal development in the shale–dominated Upper Devonian Catskill Delta Complex, western New York State. Mar. Pet. Geol..

[37] Tang X (2015). The effect of the variation in material composition on the heterogeneous pore structure of high maturity shale of the Silurian Longmaxi formation in the Southeastern Sichuan Basin, China. J. Nat. Gas Sci. Eng..

[38] Li J (2015). Key factors controlling the gas adsorption capacity of shale: A study based on parallel experiments. Appl. Geochem..

[39] Liu X, Xiong J, Liang L (2015). Investigation of pore structure and fractal characteristics of organic-rich yanchang formation shale in central China by nitrogen adsorption/desorption analysis. J. Nat. Gas Sci. Eng..

[40] Chen J, Xiao XM (2014). Evolution of nanoporosity in organic-rich shales during thermal maturation. Fuel.

[41] Yang Y, Aplin AC (2010). A permeability–porosity relationship for mudstones. Mar. Pet. Geol..

[42] Zhang X (2013). Sealing mechanism for cap beds of shallow-biogenic gas reservoirs in the Qiantang River incised valley, China. Cont. Shelf Res..

[43] Meng L (2014). Internal structure and sealing properties of the volcanic fault zones in Xujiaweizi Fault Depression, Songliao Basin, China. Pet. Explor. Dev..

[44] Wu CJ (2017). Pore characteristics differences between clay-rich and clay-poor shales of the Lower Cambrian Niutitang Formation in the Northern Guizhou area, and insights into shale gas storage mechanisms. Int. J. Coal Geol..

[45] Fan M (2011). Evaluation standard of mudstone caprock combining specific surface area and breakthrough pressure. Pet. Geol. Exp..

